# Establishment of Baculovirus-Expressed VLPs Induced Syncytial Formation Assay for Flavivirus Antiviral Screening

**DOI:** 10.3390/v10070365

**Published:** 2018-07-11

**Authors:** Shiyu Dai, Yanfang Zhang, Tao Zhang, Bo Zhang, Hualin Wang, Fei Deng

**Affiliations:** 1 State Key Laboratory of Virology, Wuhan Institute of Virology, Chinese Academy of Sciences, Wuhan 430071, China; 15629039156@163.com (S.D.); Zhangyf@wh.iov.cn (Y.Z.); tzhang@wh.iov.cn (T.Z.); zhangbo@wh.iov.cn (B.Z.); 2 University of Chinese Academy of Sciences, Beijing 100049, China

**Keywords:** baculovirus expression system, virus like particle, flavivirus, zika virus, syncytia

## Abstract

The baculovirus-insect cell expression system has been widely used for heterologous protein expression and virus-like particles (VLPs) expression. In this study, we established a new method for antiviral screening targeting to glycoprotein E of flaviviruses based on the baculovirus expression system. ZIKV is a mosquito-borne flavivirus and has posed great threat to the public health. It has been reported that ZIKV infection was associated with microcephaly and serious neurological complications. Our study showed that either ZIKV E or prME protein expressed in insect cells can form VLPs and induce membrane fusion between insect cells. Therefore, the E protein, which is responsible for receptor binding, attachment, and virus fusion during viral entry, achieved proper folding and retained its fusogenic ability in VLPs when expressed in this system. The syncytia in insect cells were significantly reduced by the anti-ZIKV-E specific polyclonal antibody in a dose-dependent manner. AMS, a thiol-conjugating reagent, was also shown to have an inhibitory effect on the E protein induced syncytia and inhibited ZIKV infection by blocking viral entry. Indeed the phenomenon of syncytial formation induced by E protein expressed VLPs in insect cells is common among flaviviruses, including Japanese encephalitis virus (JEV), Dengue virus type 2 (DENV-2), and tick-borne encephalitis virus (TBEV). This inhibition effect on syncytial formation can be developed as a novel, safe, and simple antiviral screening approach for inhibitory antibodies, peptides, or small molecules targeting to E protein of ZIKV and other flaviviruses.

## 1. Introduction

The baculovirus-insect cell expression system (BES) is one of the most widely used eukaryotic systems for heterologous protein expression and has been used for functional studies, vaccine preparations, and diagnostics. Typical properties of baculoviruses, such as the high level of very late gene expression and limited host range (namely for insects), make them highly suitable as vectors for foreign gene expression. Lepidopteran insect cell lines can grow in serum-free media and do not require CO_2_, making scale up of protein production feasible. Upon infection with a recombinant baculovirus carrying the foreign gene of interest, host cells often express the heterologous protein at very high yield and allow eukaryotic-type post-translational modifications (like N- and O-glycosylation, acylation, and phosphorylation) in contrast to prokaryotic expression systems [1]. These advantages make it possible to express proteins that are soluble and conformationally, immunogenically, and functionally similar to their natural counterparts. Thus, the BES has been broadly used for the development of subunit vaccines, including virus-like particles (VLP)-based vaccines [2,3]. A licensed human papillomavirus-like particle vaccine Cervarix^®^ has been manufactured using this system [4].

Flaviviruses are important human pathogens that cause widespread infections [5]. The recently re-emerging Zika virus (ZIKV) epidemic recognized in 2015 abruptly aroused international concern for this group of viruses [6], even though the mortality risk of many flavivirus infections is relatively low. Prior to 2007, ZIKV caused only sporadic infections with negligible clinical manifestation, but the recent outbreak of virus was linked to the development of serious neurological complications, such as microcephaly and Guillain–Barre syndrome and has caused global concern [7,8]. The virus is likely to spread more because, in addition to the main transmission by mosquito bites, it also could be transmitted by blood transfusions, sexual contact, and breastmilk. However, no specific prevention and treatment are available and there is an urgent need for the development of effective prophylactic or therapeutic agents to prevent further infections. 

ZIKV is closely related to other flaviviruses including dengue virus (DENV), Japanese encephalitis virus (JEV), and tick-borne encephalitis virus (TBEV) in terms of genome and structure [9]. Flaviviruses are spherical virions composed of a nucleocapsid core that contains a single-strand, positive-sense RNA genome, surrounded by a host-derived membrane consisting of the viral glycoproteins, premembrane/membrane (prM/M), and envelope (E). Infection by flavivirus occurs via an initial interaction of the virus with cell surface receptors, followed by internalization of the virus by endocytosis. In the mildly acidic compartments of the endosome, the transmembrane (TM) protein E mediates the membrane fusion, delivering the viral RNA into the cytoplasm and initiating virus infection. The new virions bud into the endoplasmic reticulum (ER) as immature particles consisting of 60 trimeric spikes of prM-E heterodimers. During transit through the Golgi, prM is cleaved by a host furin-like protease to become infectious mature particles composed of 180 copies of E protein and M protein forming 90 E dimers, which completely cover the viral surface [10].

The entry of enveloped viruses into host cells is mediated by envelope fusion proteins (EFPs) [11]. For flavivirus, the E glycoprotein mediates viral assembly, receptor binding, and is essential for the subsequent membrane fusion involved in viral entry upon exposure to low pH in the endocytic pathway. It also represents a major target for neutralizing antibodies, which play a critical role in protection against flaviviruses. Thus, the E protein can serve as an important target for the development of antiviral drugs and vaccines against ZIKV and other flaviviruses [10]. Flavivirus E protein presents 3 distinct domains: domain I, II, and III. At the distal end of domain II, there is a highly conserved fusion loop, which is responsible for the membrane fusion between virus membranes and cell membranes during virus entry. The C-terminal domain III displays an IgG-like fold hypothesized to contribute to viral attachment and a number of potent flavivirus type-specific mAbs have been found to target this domain. Stem and transmembrane regions are found at the C-terminal end of the E ectodomain [12].

In this study, we expressed the E protein of ZIKV and other flaviviruses, including DENV-2, JEV, and TBEV in BES. The baculovirus-expressed flavivirus E proteins in Sf9 cells retained its fusogenic ability to induce syncytial formation which is a cytopathic effect (CPE) characteristic of flavivirus infection [13,14]. Based on this phenomenon, we established an approach for the rapid and safe screening of inhibitors, such as inhibitory peptides, antibodies, or molecules, which target E protein related viral attachment or membrane fusion processes of ZIKV. This method is also potential to screen broad-spectrum flavivirus antiviral reagents, which might be especially attractive in the current situation where other important human pathogenic flaviviruses, such as DENV infection may be responsible for the complications in the recent ZIKV outbreak. Moreover, baculovirus-expressed E protein may be useful for different medical applications, from improved diagnosis of the diseases to source of antigens for the development of a subunit vaccine.

## 2. Materials and Methods

### 2.1. Cells and Viruses

Vero, 293 T, and BHK21 cells were obtained from the American Type Culture Collection (ATCC, Manassas, VA, USA), and were grown in Dulbecco’s modified Eagle’s medium (DMEM) supplemented with 10% fetal bovine serum (FBS; Gibco, Grand Island, NY, USA) at 37 °C in 5% CO_2_. *Spodoptera frugiperda* Sf9 cells were cultured in Grace’s insect medium (Gibco, Grand Island, NY, USA), pH 6.0, supplemented with 10% FBS at 27 °C. ZIKV strain SZ-WIV01 (GenBank accession no.: KU963796) was isolated from a patient who returned from Pacific Samoa [15] and obtained from Microorganisms & Viruses Culture Collection Centre (MVCCC) of Wuhan Institute of Virology, CAS (Wuhan, China). The virus was propagated in Vero cells and stored at −80 °C. An attenuated JEV strain SA 14-14-2 (GenBank accession no.: KY683775) was propagated in BHK21 cells.

### 2.2. Antibodies and Reagents

Polyclonal antibodies (pAb) against E protein and prM protein of ZIKV, E protein of JEV, GP64, and VP39 of baculovirus were used as previously described [16,17,18]. The pAbs were purified by a Protein A-Sepharose^TM^ column (Sigma-Aidrich, Darmstadt, Germany). Mouse anti-Flavivirus group antigen antibody (2A10G6) was kindly provided by Dr. Chengfeng Qin (Beijing Institute of Microbiology and Epidemiology, Beijing, China). Mouse antibodies against HA, Strep, and V5-tag were purchased from Sigma (St. Louis, MO, USA). Goat anti-mouse and anti-rabbit IgG-fluorescein isothiocyanate (FITC; Abcam, Cambridge, UK) were used as the secondary antibodies in the immunofluorescence assays (IFA). Thiol-conjugating reagent 4-acetamido-4′-maleimidylstilbene-2,2′-disulfonic acid (AMS) was purchased from Invitrogen (Carlsbad, CA, USA). Proteasome inhibitor MG1332 was kindly provided by Dr. Leike Zhang (Wuhan Institute of Virology, Chinese Academy of Sciences).

### 2.3. Construction of Recombinant Baculoviruses and Plasmids

**Figure 1:**
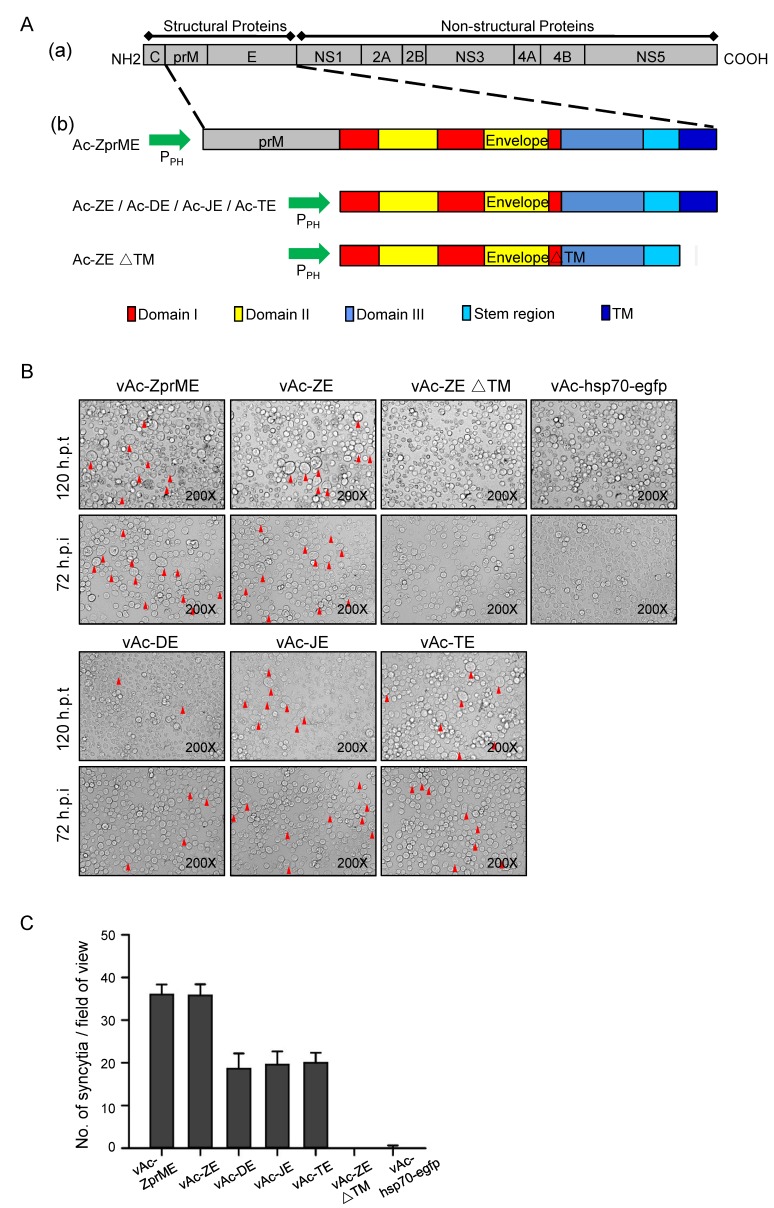
Induction of syncytial formation by baculovirus-expressed flavivirus E proteins. (**A**) Construction of recombinant baculoviruses. (**a**) Schematic representation of the flavivirus polyprotein; (**b**) Structure of recombinant bacmids expressing prME, E, and ectodomain of E (EΔTM) of specific virus. The fragments were inserted into AcMNPV bacmid under the control of a polyhedrin promoter. In the schematic diagram of domain organization for flavivirus E protein, domain I (red), domain II (yellow), and domain III (blue). A stem region links the stably folded E ectodomain with the C-terminal transmembrane anchor. (**B**) Morphology of Sf9 cells at 120 h after transfection with Ac-ZprME, Ac-ZE, Ac-DE, Ac-JE, and Ac-TE, Ac-ZEΔTM, or Ac-hsp70-efp and 72 h after infection with vAc-ZprME, vAc-ZE, vAc-DE, vAc-JE, vAc-TE, vAc-ZEΔTM, or vAc-hsp70-egfp. The arrows indicate the syncytia. (**C**) The number of syncytia within a field of view was counted for statistical analysis. The means and standard deviations (SD) correspond to data collected from five random fields of view.

The genomic RNA of the flaviviruses (ZIKV: strain SZ-WIV01; JEV: strain SA 14-14-2) was extracted from infected Vero cells using TRIzol regent (Invitrogen, Carlsbad, CA, USA), and subsequently reverse transcribed using Moloney Murine Leukemia Virus (M-MLV) Reverse Transcriptase (Promega, Madison, WI, USA) according to the manufacturer’s instructions. A plasmid pCDNA3.1-DV2prME which contains the prME fragment of DENV-2 (strain TSV01, GenBank accession no.: AY037116) was used as a template for the amplification of E fragment of DENV-2. The cDNA encoding the E protein of TBEV (strain MDJ-02, GenBank accession no.: JF316707) was obtained by gene synthesis (GS, Wuhan, China). The resultant ZIKV cDNA was used as a template for the amplification of gene fragments coding for prME, the full length of E, and the ectodomain of E (EΔTM) into a pFastBac^TM^ Dual vector (Invitrogen, Carlsbad, CA, USA) under the polyhedrin promoter (P_PH_), generating pFBD-ZprME, pFBD-ZE, and pFBD-ZEΔTM (Figure 1). The constructs were used to generate the corresponding baculoviruses, namely vAc-ZprME, vAc-ZE, and vAc-ZEΔTM using the Bac-to-bac system (Invitrogen, Carlsbad, CA, USA) according to the manufacturer’s instructions. Similarly, recombinant baculoviruses expressing E protein of DENV-2, JEV, and TBEV were constructed using the same strategies and designated vAc-DE, vAc-JE, and vAc-TE. The E protein of TBEV was fused expressed with an HA-tag at the C-terminal for detection. The baculovirus vAc-hsp70-egfp was previously constructed in our laboratory [17] and was used as a control baculovirus. To visualize the expression of E protein of ZIKV, DENV, JEV, and TBEV in the absence of virus infection in insect and mammalian cells, fragments encoding E proteins were cloned into expression vector pIZ/V5-pHsp70 vector which was constructed as described before [16] and pCAGGS with strep-tag, to generate transient expressing vector pIZ-ZE, pIZ-DE, pIZ-JE, pIZ-TE, pCAGGS-ZE, pCAGGS-DE, pCAGGS-JE, and pCAGGS-TE. All the expression plasmids were constructed by standard molecular biological approaches.

### 2.4. Cell Transfection

Sf9 cells were transfected with the indicated amounts of expression plasmids using Cellfectin (Invitrogen) according to the manufacturer’s instructions. At 48 h post transfection (h p.t.), cells were collected, and transfection efficiency was measured with immunofluorescence assay. 293 T Cells were transfected with the indicated amounts of expression plasmids using Lipofectamine 3000 (Invitrogen) according to the manufacturer’s instructions. At 18 h p.t., cells were treated with low-pH DMEM medium (pH 6.0) for 30 min at 37 °C and then incubated with DMEM medium containing 10% FBS. The multinuclear cells were detected using IFA at 18 h post-treatment.

### 2.5. Production and Purification of ZIKV VLPs

The VLPs and ZIKV virions were purified by sucrose gradient ultracentrifugation as previously described [18]. Briefly, Sf9 cells infected with the recombinant baculovirus vAc-ZE or vAc-ZprME at a multiplicity of infection (MOI) of five. At three days post infection (d p.i.), culture supernatants were collected, cleared of cell debris and then concentrated using a 20% sucrose cushion at 150,000 g (SW41 rotor; Beckman, Brea, CA, USA) for 3 h. The pellets were resuspended in NTE buffer (120 mM NaCl, 10 mM Tris-HCl and 1 mM ethylenediaminetetraacetic acid [EDTA], pH 7.5) and applied to a continuous sucrose gradient (10–60%). Following ultracentrifugation at 150,000 *g* (SW41 rotor; Beckman) for 3 h, 12 fractions (from top to bottom) were collected for subsequent Western blot analysis and transmission electron microscopy (TEM) assays.

### 2.6. TEM and Immune-Electron Microscopy (IEM)

To observe VLPs within cells, Sf9 cells were infected with vAc-ZprME, vAc-ZE, vAc-DE, vAc-JE, and vAc-TE at an MOI of five. Infected cells were harvested at 72 h post infection (h p.i.) and processed for electron microscopy as previously described [18]. Sf9 cells infected with vAc-hsp70-egfp were used as control.

After purification, the ZIKV VLPs or ZIKV particles were adsorbed onto formvar-coated copper grids for 5 min, negatively stained using 2% phosphotungstic acid (PTA) for 1 min and then examined with a transmission electron microscope (H-7000 FA; Hitachi, Tokyo, Japan).

For IEM, purified particles were adhered onto carbon-coated nickel grids (200 mesh). After blocking with 5% bovine serum albumin (BSA; Biosharp, Hefei, China), the grids were incubated with anti-ZIKV-E pAb or rabbit pre-immune serum and subsequently with the 12-nm Colloidal Gold-AffiniPure Goat Anti-Rabbit IgG (Jackson ImmunoResearch, West Grove, PA, USA). Then, the grids were negatively stained and examined with TEM as described above.

### 2.7. Western Blot Analysis of Protein Expression

Protein samples were subjected to sodium dodecyl sulfate polyacrylamide gel electrophoresis (SDS-PAGE) and transferred to polyvinylidene difluoride (PVDF) membranes (Millipore, Billerica, MA, USA). After blocking with Tris-buffered saline (TBS) containing 5% nonfat milk, the membranes were incubated with primary antibodies at 4 °C overnight and subsequently with the corresponding secondary antibodies conjugated with horseradish peroxidase (HRP) (Sigma, St. Louis, MO, USA) for 1 h at 37 °C. Protein band signals were detected using SuperSignal West Pico Chemiluminescent Substrate (Thermo Scientific, Rockford, IL, USA).

### 2.8. Immunofluorescence Assay

Transfected or infected cells were fixed with 4% paraformaldehyde-PBS and incubated in 0.2% Triton X-100-PBS for permeabilization or not, and they were then blocked with 5% BSA. The cells were then treated with primary antibodies for 1 h at room temperature and stained with corresponding secondary antibodies for 1 h at room temperature. For visualization of the nuclei, the cells were incubated with Hoechst 33258 (Beyotime, Shanghai, China) for 5 min at room temperature. Images were gained and analyzed by fluorescence microscopy.

### 2.9. Cytotoxicity Assay

Monolayers of cells in 96-well plates were treated with escalating concentrations of AMS or MG132. After 24 h of treatment, cells were assayed for viability using Cell Counting Kit-8 (CCK-8, Dojindo, Japan) according to manufacturer’s protocol.

### 2.10. Syncytial Formation Inhibition Assay

Sf9 cells were seeded onto 96-well plates at 1 × 10^5^ cells per well and infected with the specified recombinant baculoviruses at an MOI of 0.1. At 48 h p.i., the cells were incubated with escalating concentrations of polyclonal antibodies (anti-ZIKV-E, anti-ZIKV-prM, and anti-GP64), AMS, and MG132. The inhibition of syncytial formation was then detected at 4 d p.i., and statistical analysis was undertaken.

### 2.11. Viral Plaque Assay

ZIKV and JEV titers were determined by plaque assay performed on Vero or BHK21 cells. Virus stocks were serially diluted and adsorbed to confluent monolayers. After 1 h of adsorption with rocking every 15 min, the inoculum was removed and cells were overlaid with semisolid medium constituted of DMEM containing 1% methylcellulose (Millipore) and 2% FBS. Cells were further incubated for 4 days and fixed with 4% paraformaldehyde for 30 min and then stained with 1% crystal violet for 30 min. The resultant plaques were counted and multiplied by the dilution factor to determine the viral titer as plaque forming units (PFU) per milliliter.

### 2.12. Assays for Antiviral Activity

To determine the inhibitory activity of AMS on flavivirus infection, a plaque reduction assay was performed. Vero or BHK21 cells (2 × 10^5^/well) were seeded in a 12-well plate the day before infection. Virus stocks (100 PFU ZIKV/JEV) were incubated with escalating concentrations of AMS and adsorbed to confluent monolayers. After 2 h of inoculation with rocking every 15 min, cells were washed three times with PBS to remove unbound virus and the inhibitory activity was then measured with plaque assay. A time-of-addition experiment was also performed for ZIKV plaque reduction assay in Vero cell. Cells were treated with escalating concentrations of AMS 2 h pre or post of virus infection and the inhibitory activity was measured as described above.

To further determine the inhibitory activity of AMS on flavivirus infection, Vero or BHK21 cells (1 × 10^5^/well) were seeded in a 24-well plate the day before infection. Then, virus (0.1 MOI, ZIKV/JEV) was mixed with equal volume of AMS serially diluted in serum-free DMEM. After incubation for 2 h at 37 °C, the mixture was added to cells. 1 h later, the culture supernatant was replaced with DMEM containing 2% FBS. At 48 h p.i., the intracellular level of E protein in infected cells was detected with IFA as described. The supernatants were collected and viral titers were determined by plaque assay. Viral RNA was extracted and analyzed by quantitative real-time polymerase chain reaction (RT-qPCR). All determinations were performed in triplicate.

### 2.13. Quantitative PCR

Cells were harvested and intracellular RNA was extracted using Trizol reagent (Promega). Then RNA was subjected to reversed transcription using PrimeScript™RT reagent Kit with gDNA Eraser (Takara, Kusatsu, Japan). Quantitative real time PCR was performed with SYBR^®^ Premix Ex Taq™ II (Takara) on StepOnePlus Real-Time PCR System (Applied Biosystems, Foster City, CA, USA) with the primers listed in Table 1. Relative quantitation was performed using the ΔΔCT method [19] with actin as an internal control and the relative fold change was calculated by normalizing to control cells.

### 2.14. Statistical Analysis

Half maximal effective concentration (EC50) values were calculated using Probit regression method by SPSS software (version number: 17.0, SPSS Inc., Chicago, IL, USA). Statistical significance was determined by student’s *t* test, with *p*-value < 0.05 considered to be statistically significant.

## 3. Results

### 3.1. Generation of Recombinant Baculoviruses Expressing Glycoproteins of Flaviviruses

The DNA fragments encoding prME, E, as well as the ectodomain of E protein (EΔTM) of ZIKV were inserted into Autographa californica multiple nucleopolyhedrovirus (AcMNPV) bacmids under the control of the polyhedron promoter (P_PH_), respectively (Figure 1A). After transfection and infection assays, the recombinant baculoviruses, vAc-ZprME, vAc-ZE, and vAc-ZEΔTM were generated and further confirmed. Recombinant baculoviruses vAc-DE, vAc-JE, and vAc-TE expressing the E protein of DENV-2, JEV, and TBEV were generated using the same way.

### 3.2. Baculovirus-Expressed Flavivirus E Protein Triggers Syncytial Formation

In both Ac-ZprME and Ac-ZE transfected and vAc-ZprME and vAc-ZE infected Sf9 cells, we observed syncytia with multi-nuclear cells which were similar to the CPE of flavivirus infection (Figure 1B). The previous researches demonstrated that certain flaviviruses, including St Louis encephalitis (SLE) virus and JEV, could establish a persistent infection in Sf9 cells and showed CPE of formation of multi-nucleated giant cells [13,14,20]. For flavivirus, the membrane fusion is mediated by E protein and the fusion loop in domain II of E protein is highly conserved. We then tested whether the phenomenon of syncytial formation induced by E protein was common for other flaviviruses and we found that syncytia were also induced by baculovirus-expressed E protein of DENV-2, JEV, and TBEV (Figure 1B,C). While the ectodomain of ZIKV E protein, which lacks the TM region, could not induced syncytia. Flavivirus E protein is a low-pH dependent membrane fusion protein. The pH of medium ranges from pH 6.0–6.5 under routine Sf9 culture conditions and the mildly acid environment provides the possibility to trigger conformational change of flavivirus E protein to mediate membrane fusion and results in the merger of the two lipid bilayers. In contrast, for the budded virus (BV) of AcMNPV, the major envelope fusion protein GP64 triggers direct fusion between the virus envelope and the cell membrane under lower pH (around 5.5) [21]. As expected, no syncytia were found in vAc-hsp70-egfp infected cells (Figure 1B), suggesting that syncytial formation was induced by the successful expression of full length flavivirus E protein not the fusion protein of baculovirus.

**Figure 2:**
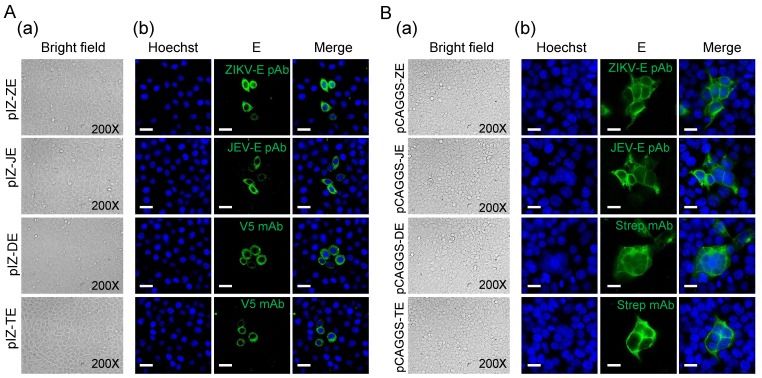
Transient expression of flavivirus E proteins. (**A**) Morphology of Sf9 cells transfected with pIZ-ZE, pIZ-JE, pIZ-DE, and pIZ-TE. (**a**) The bright field of transfected cells at 48 h p.t.; (**b**) E protein expression was detected by IFA at 48 h p.t. E proteins (green) were detected with indicated antibodies. Nuclei were stained with Hoechst as shown in blue. Bars, 10 μm. (**B**) Morphology of 293 T cells transfected with pCAGGS-ZE, pCAGGS-JE, pCAGGS-DE, and pCAGGS-TE. At 18 h p.t, cells were treated with low pH (6.0) DMEM for 30 min and then cultured using DMEM (normal pH) for 18 h. (**a**) The bright field of transfected cells 18 h after low pH treatment; (**b**) E protein expression was detected by IFA 18 h after low pH treatment. E proteins (green) were detected with indicated antibodies. Nuclei were stained with Hoechst as shown in blue. Bars, 10 μm.

In order to further exclude the possibility of a synergistic effect of baculovirus proteins on syncytial formation, plasmids expressing flavivirus E protein alone were used to transfect Sf9 and 293 T cells. In the absence of virus infection, we did not observe clear syncytia in Sf9 cells transiently expressing the E protein due to the low transient efficiency and expression level compared to those obtained following infection with recombinant baculoviruses (Figure 2A). We got similar results in mammalian cells. Under low-pH (6.0) conditions, it was also difficult to find obvious syncytia in 293 T cells transiently expressing the E protein. When analyzed using IFA, flavivirus E protein transient expressed in 293 T cells induced few syncytia (Figure 2B), indicating E protein triggered in low pH indeed can mediate membrane fusion. But the syncytia were significantly fewer than those induced by baculovirus-expressed E protein due to the low expression level.

The results suggested that flavivirus E proteins were expressed in a large quantity based on BES and retained the fusogenic ability as expressed in mammalian cells to induce syncytial formation.

### 3.3. Localization of Flavivirus E protein in Sf9 Cells

**Figure 3:**
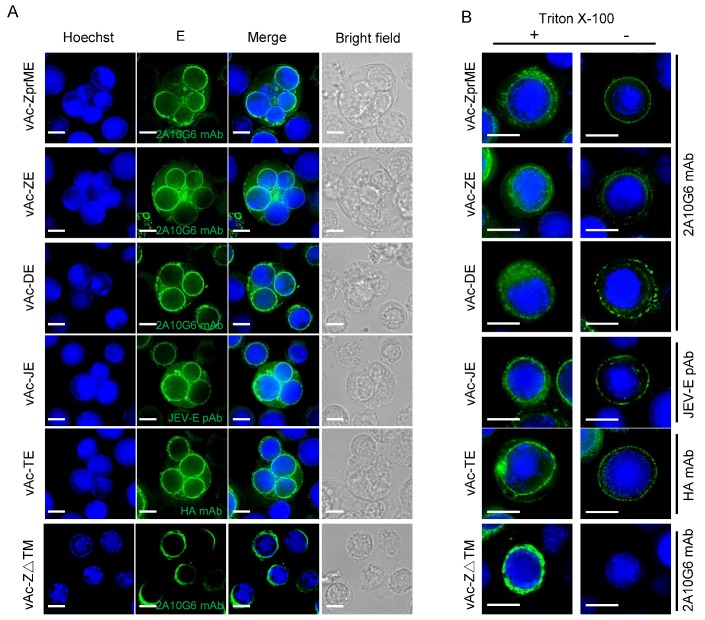
Subcellular localization of baculovirus-expressed flavivirus E proteins in Sf9 cells. (**A**) Sf9 cells were infected with vAc-ZprME, vAc-ZE, vAc-DE, vAc-JE, vAc-TE, and vAc-ZEΔTM at an MOI of 5. E proteins (green) were detected with indicated antibodies. Nuclei were stained with Hoechst as shown in blue. Bars, 5 μm. (**B**) Cell-surface expression of E protein analyzed by immunofluorescence. Cells were permeabilized with Triton X-100 (left panels) or not treated (right panels), and immunostained with indicated antibodies. Bars, 5 μm.

To investigate the underlying mechanisms of syncytia induced by baculovirus-expressed flavivirus E proteins, the subcellular localization of E protein in Sf9 cells was detected by IFA. Sf9 cells infected with vAc-ZprME, vAc-ZE, vAc-ZEΔTM, vAc-DE, vAc-JE, and vAc-TE were stained with specific antibodies. The fluorescent images showed that the E proteins in Sf9 cells infected with vAc-ZEΔTM showed an endoplasmic reticulum (ER)-like distribution. However, in vAc-ZprME, vAc-ZE, vAc-DE, vAc-JE, and vAc-TE infected cells, except for the cytoplasmic localization, a large portion of E protein was also localized to cell surface (Figure 3A).

To confirm the cell surface expression of flavivirus E proteins, fixed but unpermeabilized cells were stained with specific antibodies. As indicated in Figure 3B, the E proteins were clearly detected in unpermeabilized cells as well as permeabilized cells. However, in vAc-ZEΔTM infected cells, E protein was not detected in cell surface. We speculated that the truncated E protein, which lacks the TM region lost the membrane-integration ability, could not anchor in the cytoplasmic membrane. Thus, our observations indicated that the flavivirus E protein tended to localize to the cell surface independent of the expression of prM, which might mediate cell to cell fusion and contribute to syncytial formation.

### 3.4. ZIKV VLPs Were Generated Using Baculovirus Expression System

The Sf9 cells infected with specified recombinant baculoviruses were harvested at 72 h p.i. In Sf9 cells infected with vAc-ZprME, vAc-ZE, vAc-DE, vAc-JE, or vAc-TE, some spherical virus-like particles ranging from 50 to 100 nm in diameter were released from the cell surface (Supplementary Figure S1). In contrast, in Sf9 cells infected with vAc-hsp70-egfp, only baculoviruses which were rod-shaped and average 30–60 nm in diameter and 250–300 nm in length budded through the plasma membrane at the surface of infected cells.

**Figure 4:**
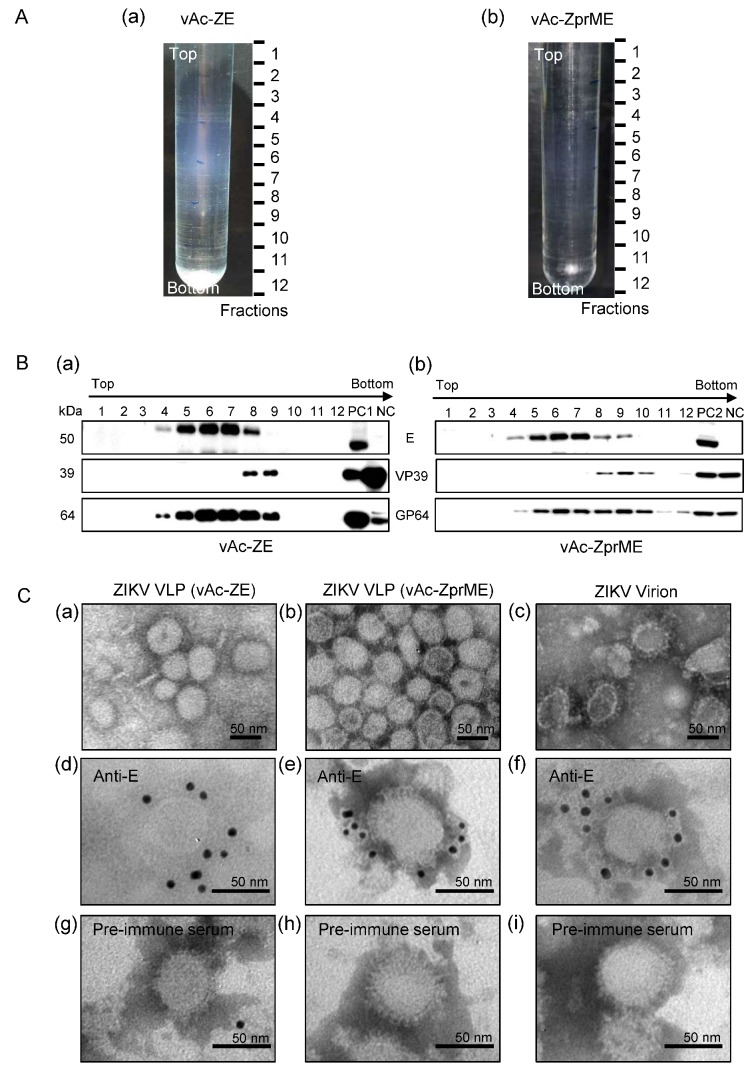
Characterization of baculovirus-expressed ZIKV VLPs from culture supernatants. (**A**) Culture supernatants of vAc-ZE (**a**) and vAc-ZprME (**b**) infected Sf9 cells were concentrated and layered onto 10–60% sucrose gradients and subjected to centrifugation. Twelve fractions were taken from top to bottom. (**B**) Western blot analysis of purified sucrose gradient fractions of culture supernatants of Sf9 cells infected with vAc-ZE (**a**) and vAc-ZprME (**b**) using the indicated antibodies. PC1: positive control 1 (vAc-ZE infected Sf9 cells); PC2: positive control 2 (vAc-ZprME infected Sf9 cells); NC: negative control (vAc-hsp70-egfp infected Sf9 cells). (**C**) Electron micrographs of negative staining and immunogold labeling of VLPs and ZIKV. (**a**,**b**) Negative staining of purified ZIKV VLPs from the E antigen-enriched fractions from the sucrose gradient fractions of culture supernatants of Sf9 cells infected with vAc-ZE (**a**) and vAc-ZprME (**b**); (**c**) Negative staining of purified ZIKV virions; (**d**–**f**) IEM of ZIKV VLPs (vAc-ZE) (**d**) ZIKV VLPs (vAc-ZprME) (**e**) and ZIKV virions (**f**) using anti-ZIKV-E-specific polyclonal antibody as primary antibody; (**g**–**i**) IEM of ZIKV VLPs (vAc-ZE) (**g**), ZIKV VLPs (vAc-ZprME) (**h**) and ZIKV virions (**i**) using rabbit pre-immune serum as primary antibody.

Supernatants of vAc-ZprME and vAc-ZE infected Sf9 cells were layered onto the 10–60% sucrose gradients and subjected to ultracentrifugation. As shown in Figure 4A,B, ZIKV E protein was distributed across the sucrose gradient, from 30% to 60%. Electron microscopic analysis of negative-stained purified ZIKV E protein rich fractions revealed rough spherical particles of 30–50 nm in size, which were similar to native ZIKV virions in morphology (Figure 4C). IEM using anti-ZIKV-E antibody further revealed immunogold particles surrounding distinct particles, which were similar to native virions (Figure 4C), indicating the exposure of E protein on the outer surface of the VLPs. All these data demonstrated that expression of ZIKV E proteins in Sf9 cells, with or without the prM protein, could self-assemble into VLPs and released from infected cells. These VLPs consisted of ZIKV E protein in the culture medium may also play a role in the syncytial formation. 

### 3.5. Syncytial Formation Was Adapted to Screen Antiviral Inhibitor

**Figure 5:**
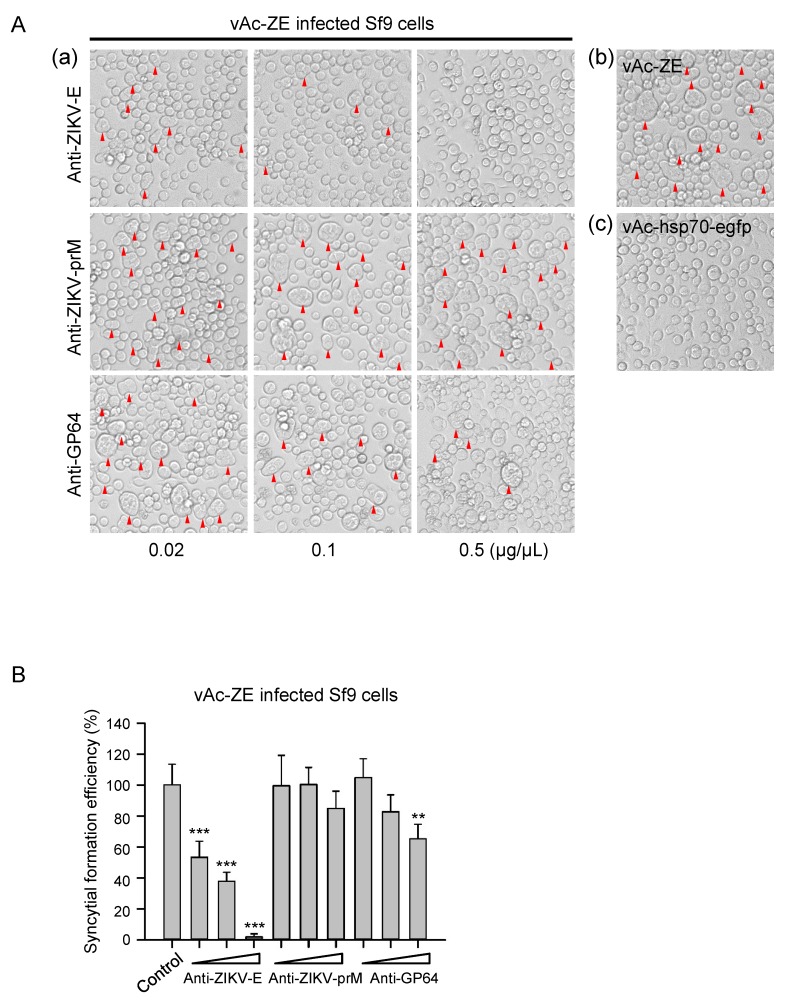
Antiviral determination of polyclonal antibodies. (**A**) (**a**) Cytopathic effect of Sf9 cells infected by vAc-ZE, with increasing concentrations of anti-ZIKV-E polyclonal antibody or unrelated polyclonal antibodies (anti-ZIKV-prM / anti-GP64) mixed in the culture medium. (**b**) Sf9 cells infected with vAc-ZE were used as a positive control. (**c**) Sf9 cells infected with vAc-hsp70-egfp were used as a negative control. (**B**) The number of syncytia within a field of view was counted for statistical analysis. The means and SD correspond to data collected from five random fields of view. Values that are significantly different from control are indicated. ** *p* < 0.01; *** *p* < 0.001.

The phenomenon of severe syncytial formation induced by baculovirus-expressed flavivirus E protein has implications in the development of a platform to screen antiviral molecules targeting the E protein of flavivirus, which is involved in receptor binding, attachment, and membrane fusion during virus entry. Primarily, the inhibition effect on syncytial formation was evaluated by taking the anti-ZIKV-E specific pAb as an example. It is demonstrated that addition of anti-ZIKV-E specific antibody could significantly reduce syncytial formation induced by ZIKV E protein in a dose-dependent manner, and the inhibition effect was almost complete at the concentration of 0.5 μg/μL (Figure 5). In contrast, in vAc-ZE infected-cells or infected-cells incubated with the antibodies unrelated to E protein (anti-prM), remarkable syncytia with multi-nuclear cells were observed. Anti-GP64 antibody which targets GP64, the envelop fusion protein of baculovirus, also had some effect on the formation of syncytia. We speculate that the inhibition of syncytia by anti-GP64 pAb was a result of the effect on the entry of baculoviruses.

**Figure 6:**
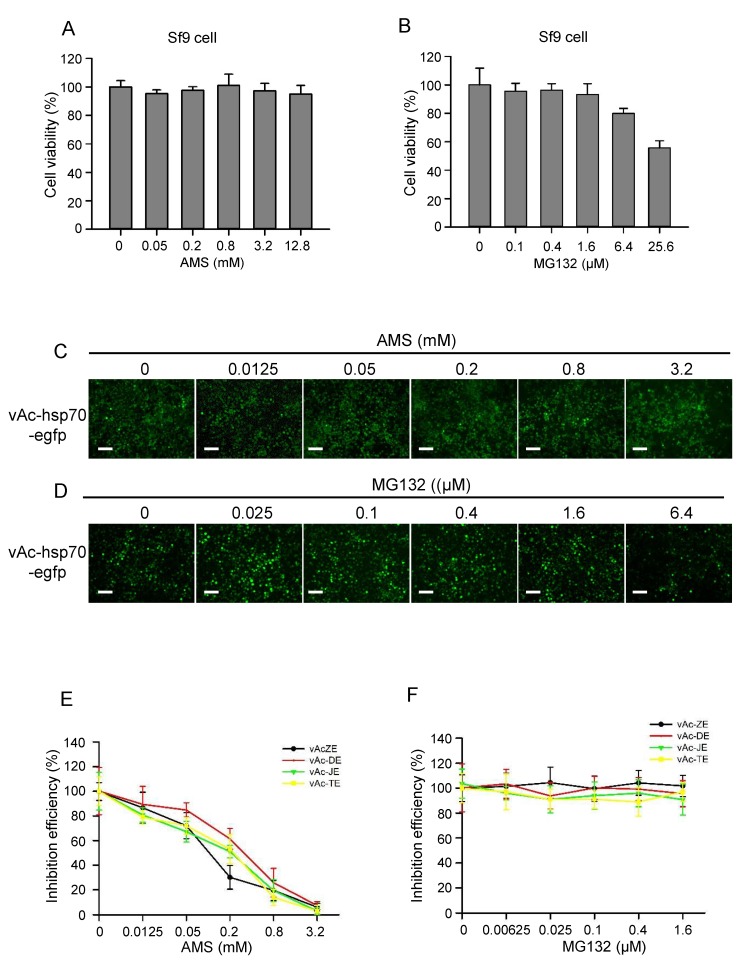
AMS but not MG132 effectively blocked baculovirus-expressed flavivirus E proteins induced syncytia. (**A**) AMS cytotoxicity was assessed by the viability of Sf9 cells treated with AMS. Pre-seeded Sf9 cells were treated with increasing concentrations of AMS for 24 h, and then subjected to cell viability analysis using CCK-8. (**B**) MG132 cytotoxicity was assessed by the viability of Sf9 treated with MG132. Pre-seeded Sf9 cells were treated with increasing concentrations of MG132 for 24 h, and then subjected to cell viability analysis using CCK-8. (**C**) The effect of AMS on the infectivity of baculovirus and expression of heterologous protein were evaluated using control baculovirus vAc-hsp70-egfp which expressed enhanced green fluorescent protein (egfp). (**D**) The effect of MG132 on the infectivity of baculovirus and expression of heterologous protein were evaluated using control baculovirus vAc-hsp70-egfp which expressed egfp. Bars, 100 μm. (**E**) AMS effectively blocked baculovirus-expressed flavivirus E proteins induced syncytia. (**F**) MG132 had no effect on baculovirus-expressed flavivirus E proteins induced syncytia. The number of syncytia within a field of view was counted for statistical analysis. The means and SD correspond to data collected from five random fields of view.

Then the other two molecules, AMS and MG132, which were reported to have effect on the entry process of flavivirus and other viruses, were also evaluated by this platform. The infectivity of many viruses is blocked by alkylation of free sulfhydryl groups on the envelope fusion proteins prior to virus attachment, as described for hepatitis C virus [22], retroviruses [23], hantaviruses [24], and hepatitis B virus [25]. Here, AMS, a membrane impermeable thiol-alkylating reagent, was used as an inhibitor targeting E protein. MG132, an inhibitor of the ubiquitin proteasome system (UPS), was reported to inhibit ZIKV infection by affecting the entry process [26]. First, the cytotoxicity of AMS and MG132 in Sf9 cells was assessed using CCK-8 (Figure 6A,B). Then Sf9 cells were infected with vAc-ZE and vAc-hsp70-egfp at an MOI of 0.1. At 2 d p.i, the cells were treated with indicated concentrations of AMS or MG132. Refer to the control baculovirus vAc-hsp70-egfp, AMS had some effect on the replication of baculovirus. But at 4 d p.i, almost all the cells were infected and the foreign protein were expressed (Figure 6C,D). AMS blocked the formation of syncytia induced by flavivirus E protein in dose-dependent manner (Figure 6E), indicating alkylation of free thiols on E protein affect its fusogenic ability to mediate membrane fusion. However, there were no significant differences between the number of syncytia of cells treated or not treated with MG132 (Figure 6F), this may be because MG132 affect ZIKV entry process by inhibiting UPS, not the E protein.

These results suggested that the system could be used for screening of antiviral molecules targeting flavivirus E protein, which may affect the entry process by inhibiting viral attachment and membrane fusion processes.

### 3.6. AMS Broadly Inhibited Flavivirus Infection by Blocking Viral Entry

**Figure 7:**
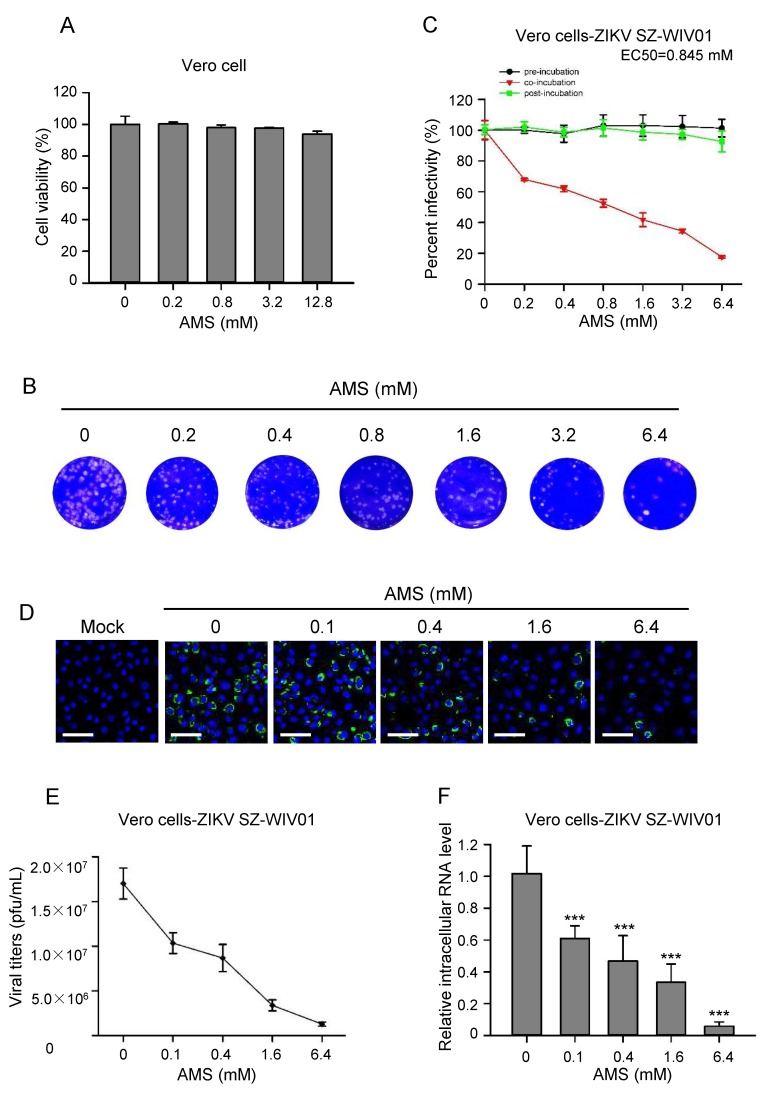
AMS affected ZIKV entry process in Vero cells. (**A**) AMS cytotoxicity was assessed by the viability of Vero cells treated with AMS. Pre-seeded Vero cells were treated with increasing concentrations of AMS for 24 h, and then subjected to cell viability analysis using CCK-8. (**B**,**C**) AMS affected ZIKV infection in Vero cells at early stage. Vero cells were treated with indicated concentrations of AMS at different time points prior, during, or after the addition of ZIKV strain SZ-WIV01 (100 pfu) and the inhibition efficiency was measured with plaque assay. The plaques of Vero cells treated with AMS during the addition of ZIKV strain SZ-WIV01 (**B**). Plaques were counted and percentage of plaque reduction was calculated (**C**). (**D**–**F**) AMS affected ZIKV strain SZ-WIV01 replication in Vero cells in a dose-dependent manner. ZIKV strain SZ-WIV01 was incubated with indicated concentrations of AMS and infected Vero cells at an MOI of 0.1. At 48 h p.i., the intracellular level of ZIKV E protein (green) in Vero cells was detected with immunofluorescence analysis (**D**). The titer of ZIKV in the supernatant was quantified by plaque assay (**E**). The intracellular level of ZIKV RNA was quantified with quantitative PCR (**F**). Bars, 50 μm. Data are means ± SD of triplicate experiments. *** *p* < 0.001.

**Figure 8:**
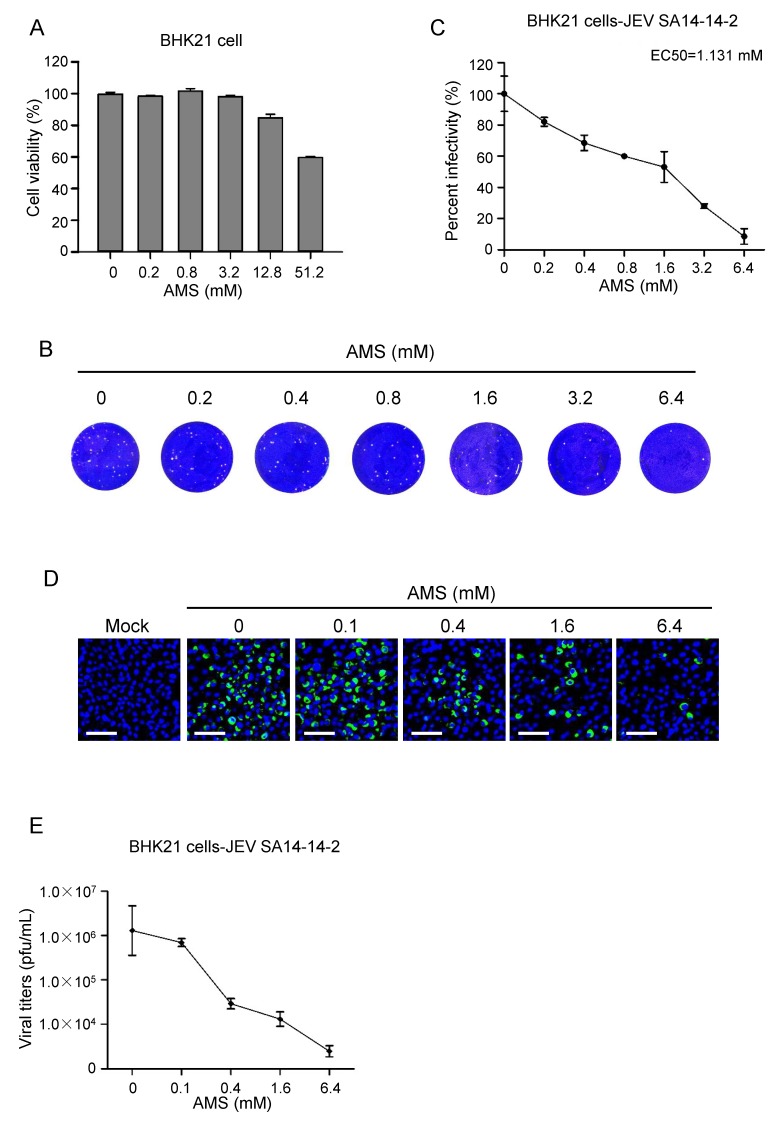
AMS inhibited JEV infection in BHK21 cells. (**A**) AMS cytotoxicity was assessed by the viability of BHK21 cells treated with AMS. Pre-seeded BHK21 cells were treated with increasing concentrations of AMS for 24 h, and then subjected to cell viability analysis using CCK-8. (**B**,**C**) Plaque reduction assay to determine the effect of AMS on JEV infection in BHK21 cells. BHK21 cells were treated with indicated concentrations of AMS during the addition of JEV strain SA-14-14-2 (100 pfu) and the inhibition efficiency was measured with plaque assay (**B**). Plaques were counted and percentage of plaque reduction was calculated (**C**). (**D**,**E**) AMS affected JEV strain SA-14-14-2 replication in BHK21 cells in a dose-dependent manner. JEV strain SA-14-14-2 was incubated with indicated concentrations of AMS and infected BHK21 cells at an MOI of 0.1. At 48 h p.i., the intracellular level of JEV E protein (green) in BHK21 cells was detected with immunofluorescence analysis (**D**). The titer of JEV in the supernatant was quantified by plaque assay (**E**). Bars, 50 μm. Data are means ± SD of triplicate experiments.

Since AMS blocked the syncytial formation induced by baculovirus-expressed flavivirus E protein, we then examined whether it had an effect on the infectivity of flaviviruses. First, the cytotoxicity of AMS on Vero cells and BHK21 cells was assessed using CCK-8. We found that AMS at concentrations as high as 12.8 mM showed no obvious cytotoxicity on Vero cells and a slightly cytotoxicity on BHK21 cells (Figure 7A and Figure 8A).

As a test of the antiviral activity of AMS, Vero cells were treated with AMS pre-, during, and post-infection of ZIKV (100 PFU), viral infectivity was measured by plaque assay. As shown in Figure 7B,C, AMS decreased ZIKV infection in a dose-dependent manner when cells were treated with AMS during virus infection and had an EC50 of 0.845 mM. However, ZIKV replication was not blocked neither in AMS, two hours pre- nor post-treated cells. These results demonstrated that AMS may inhibit infection of ZIKV before its entry into the target cells and the alkylation of the protein of ZIKV virus not the cell membrane play the key role. Vero cells were also inoculated with escalating doses of AMS-labelled ZIKV at an MOI of 0.1. At 48 h p.i., the level of E protein expression, extracellular viral titers, and intracellular ZIKV replication were measured. In line with the data from the plaque reduction assay, the result from the immunofluorescence staining showed that AMS decreased ZIKV E protein expression in a dose-dependent manner in Vero cells (Figure 7D). These results were also confirmed by the measurement of extracellular ZIKV titers and intracellular level of ZIKV RNA (Figure 7E,F). We then tested the inhibiting effects of AMS on ZIKV using BHK21 cells and found AMS decreased the infectivity of ZIKV in a dose-dependent manner and had an EC50 of 1.25 mM (Supplementary Figure S2).

The above results were performed with ZIKV, we further evaluated the antiviral activity of AMS against other flavivirus members. As shown in Figure 8, AMS was also effective in inhibiting infection of JEV in BHK21 cells with EC50 of 1.131 mM, suggesting that AMS possessed broad inhibitory activity against flaviviruses.

## 4. Discussion

The baculovirus-insect cell expression system has been well characterized for the production of recombinant proteins and has been successfully applied in life science research and the production of pharmaceutical agents. For flaviviruses, this system has been used for VLPs production of JEV [16], functional analysis of prM protein of JEV [27], and evaluation of antigenic activity of E protein of Yellow fever virus (YFV) [28]. 

Our study showed that the E protein of ZIKV was expressed successfully in a large quantity based on the baculovirus insect cell expression system. Either prME or E protein of ZIKV expressed in recombinant baculovirus infected Sf9 cells induced syncytial formation and this phenomenon was common among flaviviruses, including DENV-2, JEV, and TBEV. During biosynthesis, the flavivirus E proteins fold co-translationally with a companion or regulatory protein prM. This heterodimeric interaction is important for the correct folding and transport of the fusion protein [11]. However, our study showed that flavivirus E protein was successfully expressed independent of prM based on baculovirus and retained the ability to mediate membrane fusion. Our previous study showed that expression of prME protein of ZIKV in Sf9 cells formed VLPs which were similar to native virions in morphology and had good immunogenicity as potential vaccine candidate [18]. In this study, we further found ZIKV E protein alone self-assembled into VLPs and these VLPs carrying the E protein in a conformation corresponding to that on native virions were also released into culture supernatant and may mediate membrane fusion resembling ZIKV.

Flavivirus infections are an increasing and probably lasting global risk. Despite the success of effective flavivirus vaccines, specifically those against JEV, TBEV, and DENV and rapid progress in the preclinical development of anti-ZIKV vaccines [29,30], testing the safety and efficacy of vaccines in humans can take a significant amount of time. Effective therapeutic interventions in flavivirus diseases are also urgently needed. Inhibiting the entry of viruses into the host cells have been found to be promising and successful targets in anti-flaviviruses drugs research [31]. The E protein of flavivirus is a class II viral fusion protein responsible for the receptor binding and membrane fusion process during the initial stages of infection [9]. Targeting the flavivirus E protein can serve as important inhibition site for halting the viral entry and this complex process affords many opportunities for the development of antiviral strategies, including inhibitory peptides [32], therapeutic antibodies [33], and small molecules [34].

Here, we developed a platform to screen antiviral agents targeting flavivirus E protein based on the phenomenon of syncytial formation induced by baculovirus-expressed VLPs. Firstly, the validity of this approach was evaluated by several polyclonal antibodies. The results demonstrated that the antibody specific to ZIKV E protein reduced syncytial formation in a dose-dependent manner, but unrelated antibodies had no effect. This method can also probably screen broad-spectrum antiviral molecules for flaviviruses and the inhibition effect on syncytia was also verified by other two agents which were reported to affect the early stage of flaviviruses. The thiol-reactive reagent, AMS, effectively blocked syncytial formation induced by baculovirus-expressed flavivirus E protein in a dose dependent manner. When ZIKV or JEV were incubated with AMS, their infectivity was degraded significantly. AMS is membrane impermeable and can alkylate the free thiols which are exposed at the viral or cytoplasmic membrane surface. Our study found that when ZIKV was incubated with AMS, E protein was potentially alkylated (Supplementary Figure S3). The change in E protein may alter its conformation, which is essential for the virus to interact with the host cell. Also, AMS may have effects on the host lipid membranes, which play several roles in viral infection, including entry. While we observed no alterations in infectivity of ZIKV when AMS was added 2 h pre- or post-infection. Our results suggest that AMS had an impact on early stage of infection of ZIKV and the mechanism by which this inhibition occurs is probably related to alkylation of ZIKV E protein to affect E protein-related receptor binding and membrane fusion processes. Precisely how AMS inhibits virus entry remains unclear and requires further study. Many characters of AMS, such as membrane impermeability and high molecular limit its use as drug for clinical treatment; nonetheless, AMS will be valuable chemical tools for probing the role of disulfide bond of E protein in flavivirus infection, which can serve as an important target for anti-flavivirus drug development. Another reagent, MG132, had no effect on syncytial formation, indicating that it affected the entry of ZIKV by inhibiting the UPS but not the E protein-related attachment or membrane fusion processes.

This antiviral screening platform is a potentially safe and simple approach for the preliminary high-throughput screening of inhibitors specific to E protein of ZIKV and other important human pathogenic flaviviruses because many advantages, such as avoiding the use of live viruses, simple procedures, and high effectiveness of the BES. Since the use of live viruses in current approach for anti-flavivirus drugs screening will increase the risk of spread of these live human pathogenic viruses and the cost of maintenance of high level biosafety laboratory operation. However, the manual method used here is time-consuming and laborsome. To make this antiviral screening platform more simple and efficient, the quantification of syncytia also can be combined with automated instruments, such as cytometry. It should be noted that, the infection process of baculovirus may also be the potential target of tested molecules. It is important to exclude the interference from baculovirus.

The formations of VLPs and syncytia based on baculovirus-expressed E protein provided here will advance the development of preventive vaccine candidates and anti-ZIKV therapeutics. Furthermore, this finding provides a model system for studying flavivirus E protein functions and has implications for other flaviviruses, such as DENV, JEV, and TBEV, many of which can cause devastating diseases.

## Figures and Tables

**Table 1:** List of primers for real-time quantitative PCR.

Primer Name	Primer Sequence (5′-3′)
Actin-F	CATCCGTAAAGACCTCTATGCCAAC
Actin-R	ATGGAGCCACCGATCCACA
ZIKV-E-F	ACTGGTAGAGTTCAAGGACGCAC
ZIKV-E-R	TTCAGGCGACATTTCAAGTGG

F, the forward primer; R, the reverse primer.

## Supplementary Materials

The following are available online at http://www.mdpi.com/1999-4915/10/7/365/s1, Figure S1: Ultrathin sections of recombinant baculoviruses expressing flavivirus E proteins-infected Sf9 cells. Figure S2: AMS inhibited ZIKV infection in BHK21 cells. Figure S3: Detection of free thiols in ZIKV E protein.
