# Supplementary material for: Establishment of Baculovirus-Expressed VLPs Induced Syncytial Formation Assay for Flavivirus Antiviral Screening
Source: Viruses. 2018 Jul 11;10(7):365. doi: 10.3390/v10070365 (PMC6071280; doi:10.3390/v10070365)
Supplement: Supplementary file 1 [file viruses-10-00365-s001.zip › viruses-317368-supplementary.docx]

Article

Establishment of Baculovirus-expressed VLPs Induced Syncytial Formation Assay for Flavivirus Antiviral Screening

**Supplementary Materials:**


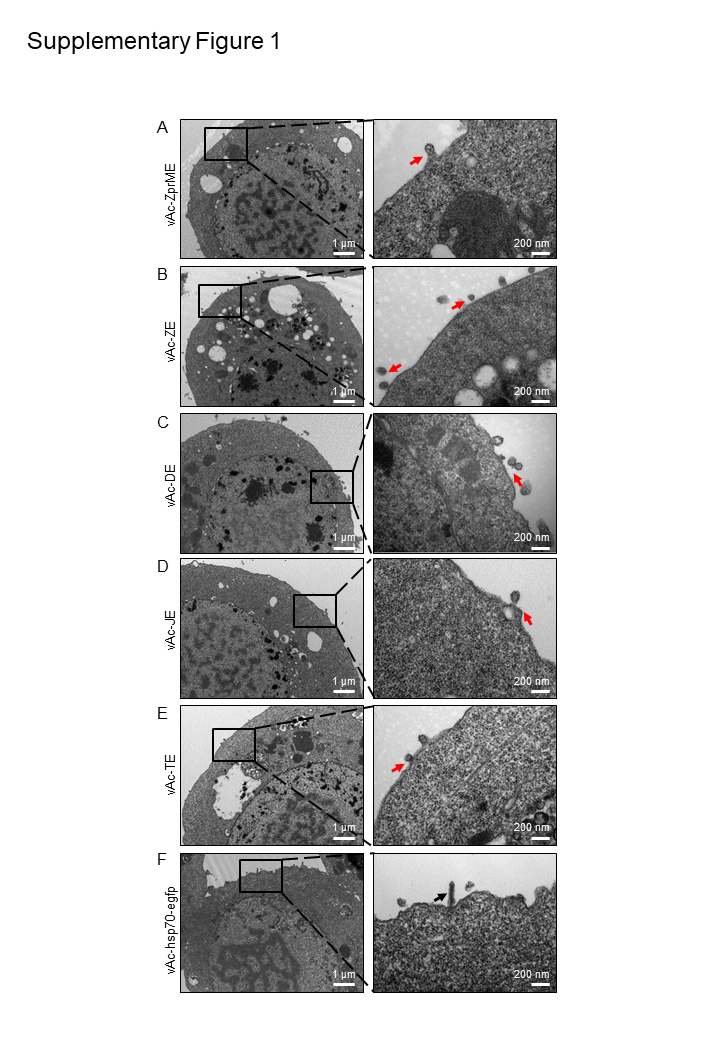


**Supplementary Figure 1.** Ultrathin sections of recombinant baculoviruses expressing flavivirus E proteins-infected Sf9 cells. (**A-E**) Transmission electron micrographs of Sf9 cells infected with vAc-ZprME (**A**), vAc-ZE (**B**), vAc-DE (**C**), vAc-JE (**D**), vAc-TE (**E**) and control baculovirus vAc-hsp70-egfp (**F**). The red arrows indicate spherical particles presumed to be VLPs which were released from the cell membrane. The black arrow indicates baculovirus particles budding from the cell membrane. The right panels show enlarged views of the boxed regions in the corresponding left panels.


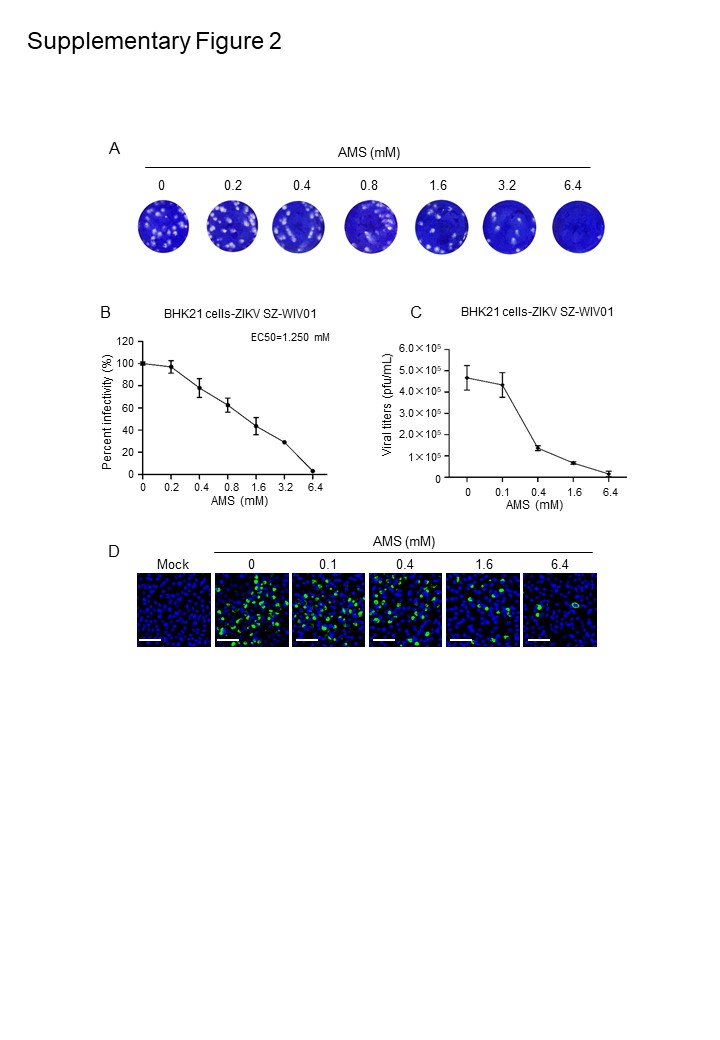


**Supplementary Figure 2.** AMS inhibited ZIKV infection in BHK21 cells. (**A,B**) Plaque reduction assay to determine the effect of AMS on ZIKV infection in BHK21 cells. BHK21 cells were treated with indicated concentrations of AMS during the addition of ZIKV strain SZ-WIV01 (100 pfu) and the inhibition efficiency was measured with plaque assay (A). Plaques were counted and percentage of plaque reduction was calculated (**B**). (**C,D**) AMS affected ZIKV strain SZ-WIV01 replication in BHK21 cells in a dose-dependent manner. ZIKV strain SZ-WIV01 was incubated with indicated concentrations of AMS and infected BHK21 cells at an MOI of 0.1. At 48 h p.i., the titer of ZIKV in the supernatant was quantified by plaque assay (**C**). The intracellular level of ZIKV E protein (green) in BHK21 cells was detected with immunofluorescence analysis (**D**). Bars, 50 μm. Data are means ± SD of triplicate experiments.


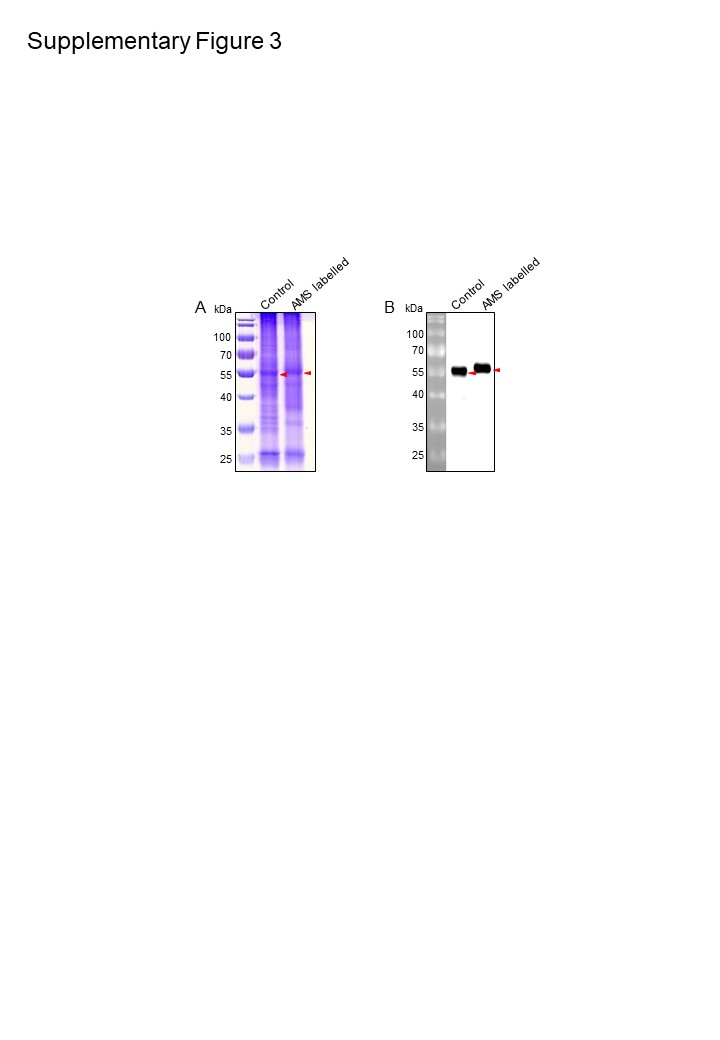


**Supplementary Figure 3.** Detection of free thiols in ZIKV E protein. (**A**) Purified ZIKV particles were labelled or unlabelled with 1mM AMS and analyzed by SDS-PAGE in the non-reducing condition. (**B**) Western blot was performed with anti-ZIKV-E polyclonal antibody. AMS can covalently react with a free thiol on the cysteine residue and increase the molecular mass by 490 D per cysteine residue. The bands corresponding to E protein were indicated by red triangles. The expected molecular weight shift of E protein is observed when ZIKV was incubated with AMS.
